# The Long-Term Algae Extract (*Chlorella and Fucus sp*) and Aminosulphurate Supplementation Modulate SOD-1 Activity and Decrease Heavy Metals (Hg^++^, Sn) Levels in Patients with Long-Term Dental Titanium Implants and Amalgam Fillings Restorations

**DOI:** 10.3390/antiox8040101

**Published:** 2019-04-16

**Authors:** José Joaquín Merino, José María Parmigiani-Izquierdo, Adolfo Toledano Gasca, María Eugenia Cabaña-Muñoz

**Affiliations:** 1 Clínica CIROM, Centro de Implantología and Rehabilitación Oral Multidisciplinaria, 30001 Murcia, Spain; jmparmi@clinicacirom.com (J.M.P.-I.); mecjj@clinicacirom.com (M.E.C.-M.); 2 Department of Neuroanatomy, Instituto Cajal (CSIC), 28002 Madrid, Spain; atoledano@cajal.csic.es

**Keywords:** algae, Chlorella, Fucus, detoxification, environmental pollution, antioxidants, heavy metals, selenium, SOD-1, neurotoxicology, aminoazuphrates, clinical medicine, nutrition, neuropathology

## Abstract

The toxicity of heavy metals such as Hg^++^ is a serious risk for human health. We evaluated whether 90 days of nutritional supplementation (d90, *n* = 16) with *Chlorella vulgaris* (CV) and *Fucus sp* extracts in conjunction with aminosulphurate (nutraceuticals) supplementation could detox heavy metal levels in patients with long-term titanium dental implants (average: three, average: 12 years in mouth) and/or amalgam fillings (average: four, average: 15 years) compared to baseline levels (d0: before any supplementation, *n* = 16) and untreated controls (without dental materials) of similar age (control, *n* = 21). In this study, we compared levels of several heavy metals/oligoelements in these patients after 90 days (*n* = 16) of nutritional supplementation with CV and aminozuphrates extract with their own baseline levels (d0, *n* = 16) and untreated controls (*n* = 21); 16 patients averaging 44 age years old with long-term dental amalgams and titanium implants for at least 10 years (average: 12 years) were recruited, as well as 21 non-supplemented controls (without dental materials) of similar age. The following heavy metals were quantified in hair samples as index of chronic heavy metal exposure before and after 90 days supplementation using inductively coupled plasma-mass spectrometry (ICP-MS) and expressed as μg/g of hair (Al, Hg^++^, Ba, Ag, Sb, As, Be, Bi, Cd, Pb, Pt, Tl, Th, U, Ni, Sn, and Ti). We also measured several oligoelements (Ca^++^, Mg^++^, Na^+^, K^+^, Cu^++^, Zn^++^, Mn^++^, Cr, V, Mo, B, I, P, Se, Sr, P, Co, Fe^++^, Ge, Rb, and Zr). The algae and nutraceutical supplementation during 90 consecutive days decreased Hg^++^, Ag, Sn, and Pb at 90 days as compared to baseline levels. The mercury levels at 90 days decreased as compared with the untreated controls. The supplementation contributed to reducing heavy metal levels. There were increased lithium (Li) and germanium (Ge) levels after supplementation in patients with long-term dental titanium implants and amalgams. They also (d90) increased manganesum (Mn^++^), phosphorum (P), and iron (Fe^++^) levels as compared with their own basal levels (d0) and the untreated controls. Finally, decreased SuperOxide Dismutase-1 (SOD-1) activity (saliva) was observed after 90 days of supplementation as compared with basal levels (before any supplementation, d0), suggesting antioxidant effects. Conversely, we detected increased SOD-1 activity after 90 days as compared with untreated controls. This SOD-1 regulation could induce antioxidant effects in these patients. The long-term treatment with algae extract and aminosulphurates for 90 consecutive days decreased certain heavy metal levels (Hg^++^, Ag, Sn, Pb, and U) as compared with basal levels. However, Hg^++^ and Sn reductions were observed after 90 days as compared with untreated controls (without dental materials). The dental amalgam restoration using activated nasal filters in conjunction with long-term nutritional supplementation enhanced heavy metals removal. Finally, the long-term supplementation with these algae and aminoazuphrates was safe and non-toxic in patients. These supplements prevented certain deficits in oligoelements without affecting their Na^+^/K^+^ ratios after long-term nutraceutical supplementation.

## 1. Introduction

Humans are exposed to pollutants, xenobiotics, and heavy metals that can be accumulated in the body when detox mechanisms are defective. Heavy metals can affect metallothionein and glutathione levels (its reduced form labeled herein as GSH) as well as SuperOxide Dismutase-1 (SOD-1) enzymatic activity [1,2,3,4]. Selenium (Se) is a crucial element for heavy metal removal by conjugation with GSH [2]. These xenobiotics can provoke hypertension and other clinical alterations in patients [5,6]. Mercury may cause neurodevelopmental disorders as autism spectrum disorders. Dental amalgams contain 50% of mercury (Hg), 41% of silver (Ag), Tin (Sn: 5–8%), Zn^++^, and Cu^++^ as minority oligoelements; titanium dental implants contain Ti-6Al-4V alloy [7]. Levels of heavy metals/oligoelements can be measured by inductively coupled plasma-mass spectrometry (ICP-MS) [8] in human samples such as urine, plasma, or hair [9,10]. The toxicity of heavy metals (e.g., mercury, cadmium) depends on the route, the concentration [11], and the exposure time and mixtures of heavy metals [12]. The function of oligoelements in odontology is still little studied (to review its functions, consult reference number [13].

The increasing concern of health problems associated with environmental pollutants is a serious one in humans because aluminum (Al) [14], lead (Pb), mercury (Hg^++^), cadmium (Cd), arsenic (As), nickel (Ni), copper (Cu^++^), iron (Fe^++^), chromium (Cr), and cobalt (Co) could provoke health problems in the case of heavy metal accumulation [14,15,16], which could be reduced by microalgae [17]. The occupational exposure to Cd and Hg^++^ are associated with antropometric activities, cremation, plastics, glass, and metal alloys. These heavy metals are also present in electrode material, nickel-cadmium batteries, water, and cigarette smoke [18,19].

Detoxification is the ability to remove drugs, mutagens, and other harmful agents from the body. The detoxification takes place in the intestinal tract, the liver, and the kidneys by microbiota able to chelate several heavy metals [20,21]. For instance, increased blood lead, mercury, and zinc levels were associated with Sarcopenia in the elderly population [6]. In addition, increased hair mercury levels (but not urinary levels) were correlated with the elevated title for the Lupus Eritematose marker in women (nuclear antigen: ANA) [10]. Several metabolic pathways in food-derived compounds are involved in detoxification [21]. Thus, clinical protocols able to prevent heavy metal accumulation are necessary in patients in conjunction with long-term nutritional supplementation. Antioxidants contribute to chelating reactive oxygen species (ROS) by removing heavy metals; thus, the screening of new antioxidants from plants is important from a clinical view point.

Some microalgae can remove heavy metals from wastewater. *Chlorella vulgaris* (CV) is a unicellular marine algae rich in chlorophyll (1–4%) that contains 55–67% protein, 9–18% dietary fiber, minerals, vitamins, and several oligoelements [17,22]. The CV algae are considered to be highly resistant to heavy metals and are widely used as a food supplement in Japan [17,23]. The *Chlorella sorokiniana* can promote antioxidant responses under zinc tolerance by increasing antioxidant enzymatic activities and increasing flavonoids, polyphenols, tocopherols, glutathione, and ascorbate (ASC) levels [24]. The CV extract can also excrete dioxin [25] and remove Cd levels by inducing metallotionein-like proteins. The biosorption of Pb^2+^ and Cd^2+^ have been described on a fixed bed column with immobilized Chlorella algae biomass [26]. *Chlorella protothecoides* algae promote heavy metal detoxification in chlordecone poisoned-treated rats by reducing the half-life of the toxin from 40 to 19 days. In addition, the *Fucus spiralis* is a marine brown alga (spiral wrack) that contains phlorotannins (antioxidant) [22]. The phytochelatins are short produced peptides from plants, algae, and fungi in response to heavy metal exposure, which detoxificate heavy metals by its high cysteine-content. *Fucus versiculosus* also may chelate Zn^++^ [22,27]. Phytochelatins are a natural source of novel angiotensin-I converting enzyme (ACE) inhibitors [28].

Our hypothesis is that the use of nasal filters (active carbon) in conjunction with long-term algae extract (*Chlorella and Fucus sp*) and aminosulphurates supplementation for 90 consecutive days contributes to the removal of heavy metals (Hg^++^, Ag, Sn, Pb) in patients with long-term dental titanium implants and amalgam fillings restorations.

## 2. Aim

We evaluated whether dietary chronic supplementation with CV and aminoazuphrates during 90 consecutive days could contribute to detoxificating heavy metals and/or prevent certain oligoelement deficits in patients with long-term dental titanium implants and amalgam fillings restorations. Therefore, the study was conducted to investigate whether long-term dietary CV contributed to the prevention of heavy metal accumulation after 90 days of supplementation (d90) in patients with long-term dental titanium implants and amalgam fillings restorations as compared with their own baseline levels (before any nutritional supplementation: d0) as well as untreated controls (without dental materials).

## 3. Materials and Methods

### 3.1. Patients

All selected patients were 49–68 years old (average: 58.5 years). The percentage of smokers was 7%, and their sociocultural states were medium-high levels (higher school education: 70%). Similar untreated (non-supplemented) control patients were included in this study. Their average age was similar to the rest of the patients. These untreated controls did not receive nutritional supplementation with the formulations. They did not have dental materials in their mouths (*n* = 21 controls).

The average number of dental amalgam fillings was 4, and there were 3 dental titanium implant alloys on average. All selected patients had a dental filling at least 10 years in their mouths (average: 15 years) and long-term titanium dental alloys for at least 10 years as well (average: 12 years). We selected 16 patients who had at least two or more long-term dental amalgams.

The number of enrolled patients suitable according to inclusion criteria was 21 untreated controls as well as 16 patients.

They received nutritional algae extracts (*Chlorella/Fucus sp*) and aminoazuphrates supplementation during 90 consecutive days (d90). Their heavy metal/oligoelement levels were compared at day 90 (d90, *n* = 16) with their basal levels (d0: before any supplementation, baseline, *n* = 16) as well as untreated controls (without dental materials in mouth, *n* = 21). These controls did not receive supplementation.

Their dental amalgams fillings were progressively replaced by composites (bisphenol A free) every 20 days following a clinical safe protocol by using active carbon filters (^@^InspiraHealth, Barcelona, Spain) and nutritional supplementation [29]. There are four quadrants in the mouth, and these amalgams were progressively replaced by each quadrant (each session within 20 days). Thus, some patients still had dental fillings during the 90 consecutive days of supplementation before their complete removal. The patients took supplements by oral intake (formulations) from the beginning (day 0, baseline levels) until the end of supplementation (d90: 90 consecutive days). We also compared levels of heavy metals/oligoelements after 90 days of supplementation with untreated controls (control, *n* = 21) and baseline levels (day zero, d0: before any supplementation). The nutritional supplementation took place during the time of dental amalgam restoration by composites. It is noteworthy that all dental materials were progressively replaced by composites at the time of collecting hair samples (90 days of supplementation). The fish consumption was 1–2 times per week in all recruited patients, including the controls. The basal heavy metals/oligoelements levels were taken at the initial visit to the dental clinic (CIROM: Centro de Implantología y Rehabilitación Oral Multidisciplinaría, https://clinicacirom.com/) before taking any nutritional supplementation, which were termed d0 (day 0) patients in the present study.

All supplemented patients received nutritional treatment by oral intake during 90 consecutive days with the following formulations: GREEN-FLOR (2-0-2; 4 capsules/day: *Chlorella and Fucus* algae extract), ERGYTAURINE (1-0-1; 2 capsule/day), and ERGYLIXIR formulations (Laboratorios Nutergia) during 90 consecutive days [from the initial day that patients visited the dental clinic (day 0) until the end of nutritional supplementation (d90: day 90)]. Controls without dental materials did not receive these treatments.

All ICP-MS heavy metal or oligoelements data were evaluated as percentiles (median, 25% and 75%) for non-parametric data (Kruskal-Wallis) and expressed as μg/g of hair in all cases (except vadanium). The ANOVA (analysis of variance) evaluated differences for vanadium levels, which were expressed as mean values ± standard error media (S.E.M). S.E.M was the variance divided by root square, and *n* was the size simple. The size sample was *n* = 16 patients at d90 and d0 and *n* = 21 untreated controls; the following heavy metals and oligoelements, respectively, were quantified by ICP-MS in the hair samples (Al, Hg^++^, Ba, Sn, Ag, Sb, As, Be, Bi, Cd, Pb, Pt, Tl, Th, U, Ni, Sn, Ti); (Ca^++^, Mg^++^, Na^+^, K^+^, Cu^++^, Zn^++^, Mn^++^, Cr, V, Mo, B, I, P, Se, Sr, P, Co, Fe, Ge, Rb, Zr).

These heavy metal/oligoelements were compared after 90 days of nutritional supplementation (d90) with their own basal levels (d0: before any supplementation) as well as untreated controls without dental materials (control, *n* = 21, non-supplemented). These untreated controls did not receive supplements and they did not carry dental materials. Saliva samples were taken at these study times for SOD-1 determination.

The limitation of the present study was the size sample (pilot study) and the absence of a placebo group. However, we included untreated controls (without dental materials and non-supplemented). This placebo group in patients with long-term dental implants and amalgam fillings could be not ethically justifiable since it is not possible to keep dental amalgam fillings, which release mercury in patients [9]. The implementation of this protocol in the Caucasian population (Spaniards) was the other limitation. 

**Figure 1:**
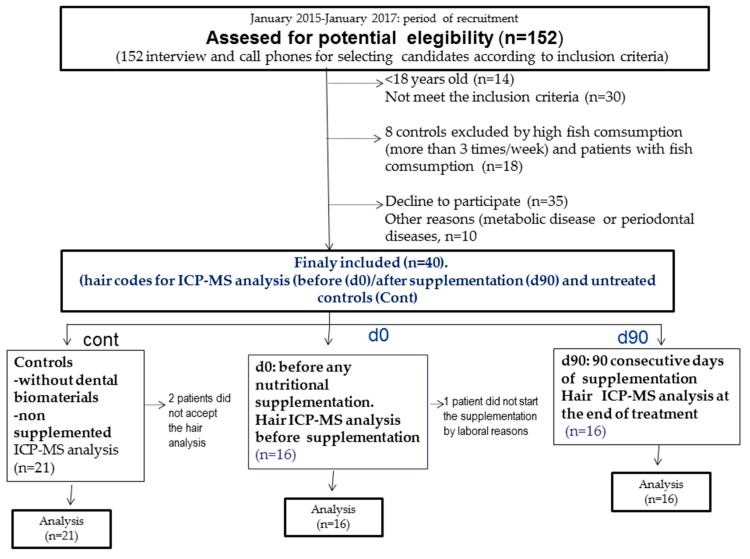
Study groups.

A dentist assessed 152 interviews or call phones to select potentially eligible patients. There were 35 patients who declined to participate and 30 patients who did not meet the inclusion criteria. We also excluded patients with fish consumption higher than 2 times by week. We did not enroll patients with periodontal disease or metabolic alterations. Thus, we selected 40 patients, and 37 participated in the end, which comprised the untreated controls (*n* = 21) and the baseline patients [before taking supplements (d0, *n* = 16) and after 90 days of nutritional supplementation (d90, *n* = 16), see study groups in Figure 1]. All statistical analyses were evaluated in 37 patients and 53 hair samples for each heavy/metal oligoelement determination here.

### 3.2. Inclusion Criteria

This study followed the Declaration of Helsinki (1974, updated 2000), and it was approved by the Institutional Review Board from CIROM (Murcia, #2016/014). All subjects were properly instructed by signing the appropriate consent paperwork. In addition, all efforts were made to protect patient privacy and anonymity. The CIROM Center was approved and certified by AENOR Spain (Spain; CIROM CERTIFICATE for dentist services, CD-2014-001 number; ER-0569/2014 following UNE-EN ISO 9001: 2008 as well as UNE 179001-2001 Directive from Spain). We selected 16 patients who had at least two or more long-term dental amalgams. They had long-term dental amalgam fillings for at least 10 years in their mouths (average: 15 years) and long-term titanium dental implants for at least 10 years (average: 12 years). The fish consumption was 1-2 times per week in all recruited patients. The average number of dental amalgam fillings was 4, and the average number of dental titanium implant alloys was 3. 

Controls were selected after clinical examination. They did not have dental materials in their mouths, nor did they show signs of periodontal diseases. We excluded patients who had fish consumption higher than 2 times by week.

### 3.3. Exclusion Criteria

Physically handicapped patients who had metabolic diseases [diabetes, metabolic syndrome, liver/kidney disease, systemic inflammation, lupus/autoimmune disease, thyroid disease, adrenal disease, or neuropsychiatry disorders Diagnostic and Statistical manual of Mental disorders (4^th^ Edition, DSM IV)] [30], were excluded in the present study. Patients taking regular medication or stimulants, anticonvulsants, atypical antipsychotic drugs, or those who had history of liver/kidney disease or DMSA (dimercaptosuccinic acid) prescribed (or chelators) patients were also excluded. Particularly, hypertensive patients and those who had periodontal disease tattoos or were taking nutritional supplements were excluded in the present study. Finally, patients who had orthodontic devices were not included here. The correct diagnostic of periodontal disease was based on several parameters, such as visual exploration (palpation), presence of dental calculus, radiographic evaluation, dental mobility, and oclusal exploration (pathological eroding facets). Periodontal disease was also a cause of exclusion, which was identified by following several criteria by an expert dentist, such as a deep dental probe higher than 3 mm, loss of bone (radiography), possible bleeding, and dental mobility [31]. 

### 3.4. Composition of Nutritional Supplementation (Algae and Other Bioactive Phytomolecules)

All patients took the following nutritional supplementation during 90 consecutive days (oral intake): GREEN-FLOR (2-0-2), ERGYTAURINE (1-0-1), and ERGYLIXIR formulation (Nutergia, 1 bottle/month) following the patterns of their antioxidants properties (see Table 1). The controls of the intakes were registered by dentists every 20 days, and we administered the following dosages according our previous clinical experience. 

### 3.5. Inductible Coupled Mass Spectromery Analysis (ICP-MS) 

In the weight of the dental amalgam fillings, mercury (Hg) was 50%, and silver (Ag) was 41%, Sn was approximately 5–8%, and Cu^++^ and Zn^++^ levels were in the minority. Hair samples close to the scalp were taken from all subjects (0.25 g from the occipital area) to measure a plethora of heavy metals/oligoelements by ICP-MS (Doctor’s Data, USA). Doctor’s Data is a pioneer laboratory specializing in the toxicology of heavy metals with over 35 years of experience, and they provide analytical tests for healthcare practitioners. ICP-MS values for heavy metals were expressed in μg/g of hair. 

### 3.6. Super Oxide Dismutase-1 (SOD-1 Activity)

The saliva SOD-1 activity was measured following a modified protocol by [9] Cabaña-Muñoz et al., 2015. Briefly, the buffer assay contained 0.1 mM EDTA (Ethylenediaminetetraacetic acid), 50 mM sodium carbonate, and 96 mM of nitro blue tetrazolium (NBT). Then, 470 µL of the above mixture was added to 100 µL of saliva, and the auto-oxidation of hydroxylamine was observed by adding 0.05 mL of hydroxylamine hydrochloride (pH 6.0). Finally, SOD-1 activity was measured by the change in optical density at 560 nm for 2 min at 30/60 s intervals and normalized as optical density (D.O) by protein [9].

### 3.7. Statistical Analysis

All data were analyzed by SPSS software (v17.0) (U.C.M: Universidad Complutense, Madrid, Spain) and Sigma Plot (v11.0, U.C.M, Madrid, Spain). Mean 25%, 75%, and median values (μg/g of hair) were estimated for heavy metals/oligoelements in the hair samples. Non-parametric tests were applied in cases without homogeneity of variance (Mann Whitney/Kruskal Wallis). The Bonferroni tests were applied for multiple comparisons when there was homogeneity of variance (e.g., vanadium, V). All results were expressed as percentiles 25%, 75%, and median (μg/g of hair) according to Kruskal Wallis values (H value) and Mann Whitney (MW) and Dunn’s post hoc test in the case of non-parametric data between (d0: *n* = 16, d90: *n* = 16) and controls (control: *n* = 21). The Levene test identified whether or not there was homogeneity of variance depending on its significance. Correlations between variables were performed by Spearman’s rank correlation. Differences were considered statistically significant if *p* < 0.05 and highly significant when *p* < 0.01.

## 4. Results

### 4.1. SOD-1 Activation Reflects Antioxidants Responses in Patients after Long-Term Supplementation with Algae Extract and Aminoazuphrates Compared with Untreated Controls

There was a statistically significant effect for SOD-1 activity in the Kruskal Wallis analysis (H = 45.1, *p* ≤ 0.001) for SOD-1 activity (saliva). The parametric Dunn’s non-analysis revealed decreased SOD-1 activity after 90 days of supplementation (d90) compared to their basal levels (d0: before any supplementation, *p* < 0.05); Conversely, increased activity SOD-1 activity was detected before any supplementation as compared with untreated controls (without dental materials, *p* < 0.05, Table 2).

### 4.2. Reduced Mercury (Hg^++^) and Silver (Ag) Levels after 90 Days of Nutritional Supplementation (d90) as Compared with Their Baseline Levels (d0) as ell as Untreated Controls (Without Dental Materials and non Supplemented, cont)

We compared levels of heavy metals (Hg^++^, Ag, Sn) as well as titanium alloys (Ti-6Al-4V) in patients with long-term dental titanium implants and amalgams fillings after 90 days of nutritional supplementation as compared with their own basal levels (before any supplementation, d0) as well with non-supplemented controls (without dental materials, controls). The Kruskal Wallis and Mann Whitney post hoc analyses revealed mercury (Hg^++^) and tin (Sn) reductions after 90 days of supplementation as compared with their own basal levels (d0) without reaching a significant effect in Zn^++^, Co, Ni, or Cu^++^ levels (Table 3).

Finally, Ag levels decreased after 90 days as compared to their basal levels (d0, before any supplementation) without reaching a significant effect as compared to untreated the controls.

The aluminium (Al) levels decreased after 90 days of supplementation (d90) as compared with untreated controls (*p* < 0.05); increased d90 vanadium (V) levels were observed as compared with basal levels (d0, *p* < 0.05). There were no effects in Ti or Co levels by treatment (*p* > 0.05, non-supplemented, Table 3). 

### 4.3. Levels of Oligoelements Involved in Metabolic Functions (Se, Mn^++^, Li, Mg^++^, Ge, S, P, I, Ca^2+^, Sr, Na^+^, K^+^)

Patients with long-term titanium implants and amalgam fillings increased germanium (Ge), manganesum (Mn^++^), chromium (Cr), vanadium (V), phosphorum (P), and lithium levels (Li) after 90 days of supplementation (day 90) as compared with untreated controls (control, *n* = 21). In addition, after 90 days (d90, *n* = 16), their selenium (Se) levels decreased in comparison to their basal levels (d0, *n* = 16, *p* < 0.05, μg/g of hair); however, they were higher than the control values (Table 4, *p* < 0.05). These supplements could promote antihypertensive effects by rising certain oligoelements. Finally, there were no effects in other oligoelements (Ca^2+,^ Mg^2+,^ I, Sr, B, Rb) or for Be, Bi, Tl, To (data not shown, *p* > 0.05, n.s).

### 4.4. Metals of Environmental Exposure

The algae extract and aminoazuphrates supplements decreased lead (Pb) levels after 90 days of supplementation (day 90) as compared with baseline levels (d0: before any supplementation); the aluminium (Al) levels were reduced after 90 days in comparison to untreated controls (see Table 5).

There was a lack of effect in several heavy metals (As, Ti, Pt, Sb, Tl, To, Cd, Be, Bi, Zr, *p* > 0.05, n.s) and oligoelements by treatment (Zn^++^, Cu^++^, Ca^++^, Sr, B, I, K^+^, Mg^++^, Rb) after 90 days of supplementation as compared with their baseline (d0) and control levels.

### 4.5. Effects on Selenium (Se) Ratios and Heavy Metals after 90 Days of Nutritional Supplementation

For example, we found decreased Se/Hg^++^, and increased Se/Al, and Mo/Hg^++^ ratios after 90 days of supplementation (d90) compared to their respective basal levels (before any treatment, d0, *p* < 0.05); However, these Se/Hg^++^, Se/Ag, and Mo/Hg^++,^ and Na^+^/K^+^ ratios decreased before any treatment (d0) as compared with non-supplemented patients (controls, *p* < 0.05, Table 6).

### 4.6. Correlations between Selenium (Se) and Heavy Metals Ratios after 90 Days of Nutritional Supplementation

The *r* Spearman correlations between selenium and heavy metal ratios are shown in Table S1. For example, there was a strong correlation between the Se/Hg^++^ (d90) ratio and Se levels after 90 days of supplementation [Se (d90), *r* = −0.76, *p* = 0.004] as well as with Mo/Hg^++^ (d90) ratio after 90 days (d90, *r* = 0.6, *p* = 0.02). Two outlier values were excluded for statistical analysis herein (Table S1, see Supplementary Materials). Table S2 showed other correlations between heavy metals and oligoelements (see Supplementary Materials). 

## 5. Discussion

This section discusses the effects of dental amalgam restoration in mercury reduction in patients with long-term titanium implants and dental amalgam restorations using carbon active (nasal filters) and long-term algae and aminoazuphrates supplementation. 

The exposure derived from amalgam fillings exceeds that from food, air, or beverages. Chronic nutritional supplementation contributes to preventing mercury release peaks caused by dental amalgam restoration (replacement by biocompatible materials like Bisphenol A free composites). A study of 12 patients demonstrated that the long-term presence of dental amalgam (at least five years) did not result in any remarkable changes in mercury or tin levels in the pulp tissue after comparing 12 restored amalgams and 12 non-restored patients. However, elevated blood mercury levels were observed even five years after the placement of the restoration [32]. These data suggest that mercury release is important even after complete dental amalgam restoration with composites, because five years after its restoration, mercury is still present in the blood [32]. Bergerow et al. reported that within 12 months after removing dental amalgam fillings (restoration by composites), patients showed substantially lower urinary mercury levels [33]. In the present study, the period of supplementation was shorter (three months: 90 days), which minimized mercury release by using carbon active (nasal filters) during dental restorations [29]. The synergic algae and aminoazuphrates treatment contributed to activating the detoxification because the mercury reached peaks shortly at 24 h after replacement with composites until 3–7 days later [34]. 

### 5.1. Detoxification of Heavy Metals in Patients with Long-Term Amalgam Fillings and Titanium Dental Implants

We determined that chronic nutritional *Chlorella* and *Fucus* algae extract supplementation in conjunction with aminosulphurates lowered certain heavy metal levels in patients with long-term titanium implants and dental amalgams restoration using activated carbon active nasal filter as well as the nutraceuticals. Preclinical findings suggest a role of *Chlorella vulgaris* as a heavy chelator in preventing toxicity of certain xenobiotics and accelerating dioxin excretion in rats [25,35]. The mercury and tin reduction after 90 days in patients agreed with enhanced heavy metal removal by *Chlorella sp* [36,37,38]. However, the exact mechanism by which chronic algae consumption removes heavy metals has not been tested yet in humans. Our aim is to develop a clinical and practical protocol to chelate heavy metals with a mixture of bioactive nutraceuticals such as algae extracts and aminosulphurates that could act independently of signaling pathways involved in detoxification. 

Supplementation with *Chlorella sp* promoted detoxification of heterocyclic amines (carcinogenic chemical) in six young Korean adults [39]. This randomized, double blind, placebo-controlled crossover study was performed in six female supplemented-patients; the nutritional period of three months in our study was longer than in the Korean study. Our findings also reflected enhanced removal of certain heavy metals, including lead (a metal of environmental exposure). Our patients’ Hg^++^, Sn, and Pb accumulations were strongly reduced after 90 days of consecutive nutritional supplementation as compared with basal levels (before any supplementation). Interestingly, mercury and Sn levels reductions were observed after 90 days as compared with untreated controls (without dental materials and non-supplemented).

### 5.2. SOD-1 Activity in Patients with Long-Term Dental Titanium Implants and Amalgams Restorations

Although it was not possible to elucidate the exact nutraceutical involved in SOD-1 activation here, we must consider that SOD-1 activation decreased after 90 days as compared with their basal levels (d0, before any supplementation). Conversely, higher SOD-1 activity was observed after long-term supplementation (day 90) compared with untreated controls; this suggests algae and aminoazuphates treatment may activate SOD-1. In addition, increased Mn^++^ levels could suggest enhanced antioxidant responses after 90 days of supplementation. In fact, Ala16Val MnSOD-2 polymorphism has been described in cells exposed to methylmercury [40]. We have previously observed higher SOD-1 activation in women with long-term dental amalgams only (without titanium dental alloys) as compared with controls (without dental materials) [9]. Our clinical findings were in consonance with the detoxification induced by Ag nanoparticles through inducing SOD, peroxidase, catalase, and glutamine synthetase enzymatic activities [41,42]. The silver (Ag) reduction after 90 days of supplementation as compared with the patients’ baseline levels (before any supplementation) agreed with the enhanced removal of heavy metals. However, silver levels after 90 days did not differ with controls. 

Heavy metals detox requires (i) a healthy gut microbiome state [20], (ii) the induced-activation of endogenous hepatic I-II-enzyme, which can be activated by phytonaturals in these formulations [43], and (iii) the chelation and excretion of these heavy metals [44]. Steps (i) and (ii) are activated by natural products from these formulations. The ERGYLIXIR formulation contains synergic depurative bioactive compounds from extracts such as *Cinara scolymus* (artichoke) [45], *Raphanus niger* [46], *Taraxacum officinale* [47], *Arctium lappa* (dandelion root) [48], *Vaccinium macrocarpo* [49], *Solidago virgaurea* (quercitin, afzelin) [50], *Rosmarinus officinallis* [51], *Scolymus hispanicus* [52], and *Sambucus nigra* (elderberry with antocianines) [53]. In addition, sulfur-rich extracts such as garlic acid (*Allium sativa* in the ERGYTAURINE formulation) may enhance heavy metal removal by inducing antioxidant activities [54,55,56]. Apple pectin [56] and acerole (very rich in vitamin C) also contribute to heavy metals removal [57]. In addition, *Sambucus nigra* (elderberry) contains antocyanines that supply 87% of the daily vitamin C levels necessary for humans [57]. Vitamins B6, B-9, and B-12, as well as Se, Zn^++^, and Mg^++^ (ERGYTAURINE formulation) [58] are necessary for certain enzymatic activities.

### 5.3. Possible Role of Selenium (Se) in Detox after Long-Term Chlorella CV Supplementation in Patients

Because Se levels decreased after 90 days of supplementation, we cannot exclude the possibility that selenomercurials reflect the Se-heavy metal complex formation in order to prevent mercury toxicity (or other metals) in patients with long-term dental amalgams and titanium alloys. As the Na^+^/K^+^ ratio did not differ after 90 days as compared with their basal levels (before any supplementation), we can confirm that chronic algae and aminoazuphrates supplementation are safe and non-toxic for humans. The increased Se/Hg^++^ ratios suggest enhanced detoxification after 90 days compared to their basal levels (before any supplementation) as well as untreated controls. Surprisingly, a toxic effect has been demonstrated in autistic children who had elevated hair selenium levels [59]. These lower Se levels observed in conjunction with the lack of effect on the Na^+^/K^+^ and Se/Pb ratios could prevent mercury accumulation at 90 consecutive days of supplementation. In fact, antagonistic interaction between selenomethionine enantiomers and methylmercury toxicity was described with *Chlorella sorokiniana* [60]. Mercury loss with *Chlorella vulgaris* is largely influenced by amino acids, cysteine being the most effective in promoting the detoxification of mercury (Hg^2+^)^−^ in *Chlorella sp* exposed to this metal [61]. The amino acid taurine (ERGYTAURINE formulation) is derived from cysteine [62] and also contributes to heavy metal detoxification. In fact, increased oxidative stress and low systemic taurine levels were demonstrated in patients with long-term dental amalgam fillings and/or titanium alloys [63]. This indirect evidence agreed with a study in which selenocystine (SeCys_2_) reduced MeHg cytotoxicity in Hepatic HepG_2_ cells by inducing MeHg-glutathione (GSH) and also formed MeHg-cysteine (Cys) complex in vitro [64]. These indirect findings suggest that selenium contributed to detoxification in the present clinical study. Uchikawa et al. (2011) described the enhanced removal of tissue methylmercury in (BP) *Parachlorella beijerinckii*-fed mice; this continuous BP intake (10%) accelerated MeHg excretion and subsequently decreased tissue mercury accumulation by inducing the GSH metabolism [65]. 

Other metals such as Pb, Cd, and U that are associated with occupational exposure were significantly decreased after three consecutive months of supplementation compared with their basal levels (before any supplementation) without affecting the untreated controls (without dental materials). The biosorption of Pb^2+^ and Cd^2+^ was detected using a fixed bed column analysis with immobilized Chlorella algae biomass [66]. Pb levels decreased after 90 days of supplementation, agreeing with the 56% Pb reduction at four days of algae *Chlorella sp* supplementation, 69% at eight days, and 77% at 12 days of treatment [26]. Although U levels were within the normal detection range in our patients, their decrease after 90 days of supplementation was crucial. As selenium-enriched spirulina formulation reduces the development radiation that is *pneumonitis*-induced [67], the lower U levels after chronic algae supplementation are important from a clinical view point. In addition, a glutathione-dependent detoxification pathway has been described in *Chlorella algae* exposed to U [68,69,70].

### 5.4. The Nutritional Supplementation after 90 Days Prevented Certain Oligoelements Deficit in Patients with Long-Term Titanium Implants and Dental Amalgam Restorations

These polyphenols from Azorean brown algae (*Fucus spiralis or Fucus vesiculosus in* GREEN-FLOR formulation) may enhance heavy metal removal in patients with long-term dental fillings and titanium alloys. In fact, the marine algae *Ulva lactuca* and *Fucus vesiculosus* can sequester Cd and Cu^++^ [70], which explained the induced-detoxification here. The phlorotannins have potential impact on public health, particularly in hypertensive patients [71,72]. The in situ determination of trace elements in fucoids by field-portable-X-ray fluorescence (FP-XRF) provides a rapid monitoring environmental contamination [73]. Increased mercury levels can provoke hypertension, and Se may exhibit a protective effect against cardiovascular disease [6]. Long-term nutritional supplementation could increase germanium (Ge) levels in patients with long-term dental amalgam fillings and titanium implants, seemingly by reflecting antihypertensive effects. However, a direct causal relationship between antihypertensive effects and Cr and Ge elevations was not conclusive in the present study. The Sn-Se correlation observed in conjunction with Ge, Li, Cr, P and I elevations after 90 days of supplementation could be explained by the high oligoelement content (10–15%) in the supplement, resulting from its marine origin [74]. The detoxification of Hg^++^ and Cd levels here agreed with the enhanced Hg^++^, Cd^++^, and Pb removal by *Fucus* from contaminated salt waters exposed to heavy metals for seven days [74]. The *Fucus sp* algae is also traditionally used to prevent obesity or gastrointestinal diseases. As *Fucus vesiculosus* extracts reduced the blood glucose peak in mice fed with a normal diet [75], the possibility that chromium Cr and Ge elevation could contribute to these antihypertensive effects should not be excluded here. These oligoelements also increased after 90 days of supplementation as compared with untreated controls. The increased Li levels suggest a better regulation of gut microbiota after treatment with these formulations, since the host serotonine biosynthesis is regulated by intestinal microbiota [76]. In fact, the strong r Spearman correlation together with the Se/Li ratio and Li correlation suggest a better state of gut microbiota in treated patients at 90 days of supplementation as compared with their basal and control levels. Finally, the augmented phosphorous (P) levels described here may have been a consequence of chronic spirulina supplementation (GREEN-FLOR). Since undernourished children receiving *Spirulina platensis* plus Misola extract treatment have a better hematocrite that those taking Misola alone [77], the chronic algae-supplementation could prevent iron deficit. These synergic supplementations contribute to heavy metal removal in these patients. Moreover, increased systemic malondialdehyde levels and lower Mo/Co and Mo/Fe^2+^ ratios have been described in patients with long-term dental titanium implants and dental amalgams [74]. Further studies should evaluate detoxification pathways by which long-term supplementation *Chlorella or Fucus vesiculosus* treatment contribute to the removal of heavy metals in patients with long-term dental amalgam fillings and titanium implants. The absence of placebo, the non-RCT (randomised controlled trials), the size sample (pilot study), as well as the Caucasian population (Spaniards) are limitations in this study. 

## 6. Conclusions

The aminosulphurates and *Chlorella and Fucus sp* algae supplementation enhanced detoxification of heavy metals by reducing Hg^++^, Ag, Sn, and Pb levels in patients with long-term dental amalgam filling and titanium implants. The chronic nutritional supplementation with algae extract reduced Hg^++^ and Sn levels in patients with long-term titanium implants and dental amalgam restorations as compared with untreated controls (without dental materials). In addition, increased Mn^++^, Li, Ge, Cr and lower U levels, and decreased Se levels were observed after 90 days of supplementation as compared to their basal levels (before any supplementation). These findings suggest that these nutraceuticals promote beneficial effects in patients. The safety of long-term algae and aminoazuphrates supplementation were confirmed by the lack of effect in Ka^+^/K^+^ and Se/Pb ratios after 90 days compared to their basal levels (before any supplementation) and untreated controls. The SOD-1 activity could explain antioxidant and enhanced detoxification of certain heavy metals by nutritional supplementation in the present study.

## Figures and Tables

**Table 1:** Composition (nutraceuticals) of formulations.

**GREEN-FLOR (Formulation-1)**	
Nutritional supplementation during 90 consecutive days (4 capsules/day 2-0-2)	dosage (mg/day)
Chlorella: 80 mg/capsule	320 mg/day
Spirulina: 80 mg/capsule	320 mg/day
Kelp of Pacific: 60 mg/capsule	240 mg/day
Fucus: 30 mg/capsule	120 mg/day
Cardille: 25 mg/capsule	100 mg/day
Pectine of apple: 60 mg/capsule	240 mg/day
Acerole (rich in vitamin C): 50 mg/capsule	200 mg/day
Fructooligosacarides: 280 mg	1120 mg/day
*Scolymus hispanicus:* 60 mg/capsule	240 mg/day
**ERGYTAURINE (Formulation-2)**	
Treatment: 90 consecutive days (2 capsules/day; 1-0-1)	dosage (mg or μg/day)
Selenium (Se): 25 μg/capsule	50 μg/day
Vitamin B6: 0.8 μg/capsule	1.6 μg/day
Folic Acid (B-9): 100 μg/capsule	200 μg/day
Zinc (Zn++): 3.5 mg/capsule	50 μg/day
Taurine: 120 mg/capsule	240 mg/day
Extract of *Raphanus niger L*. 15 mg/capsule	30 mg/day
**ERGYLIXIR (Formulation-3)**	
Extracts from:	dosage: mg/month
*Cynara scolymus* (artichoe)	1440 mg
*Raphanus niger*	900 mg
*Taraxacum officinale*	400 mg
*Arctium lappa*	320 mg
*Vaccinium macrocarpo*	228 mg
*Solidago virgaurea*	200 mg
*Rosmarinus officinalis*	640 mg
*Sambucus niger* (elderberry: antocianines)	200 mg
Sodium Moligdate	50 μg
Selenium	50 μg

**Table 2:** Regulation of SuperOxide Dismutase-1 (SOD-1) by long-term algae and aminosulphurate supplementation and SOD-1 activity.

**Group**	**Median**	**25%**	**75%**
SOD-1 activity (control)	100	100	100
SOD-1 activity (d0)	143.5	139.500 *	149
SOD-1 activity (d90)	121	119.000 *,#	125
**SOD-1 Activity**	**Difference of Ranks**	**Q**	***p* < 0.05?**
Control vs. d0 (before treatment)	34.000	6.7	Yes
d0 vs. d90 (after 90 days)	16.000	2.920	Yes
d90 vs. Cont: controls	18.500	3.600	Yes

* *p* < 0.05 vs. control, # *p* < 0.05 d90 vs. d0; control: controls without dental materials and non-supplemented (*n* = 21); d0: patients with long-term titanium implants and dental amalgam fillings restorations before any nutritional supplementation (d0, *n* = 16); d90: patients with long-term titanium implants and dental amalgam fillings restorations after 90 days of supplementation (d90, *n* = 16).

**Table 3:** Heavy metals/oligoelements of dental materials.

**Heavy Metals from Dental Amalgams**				
**Hg++**	**Median**	**25%**	**75%**	**H**
Control	1.6	1.25	2.3	H = 13.85, *p* < 0.001.
d0	1.9	1.9	3.7 *	MW (* *p* < 0.005).
d90	1.15	0.34	2.1 *,#	MW (# d90 vs. d0, *p* = 0.049); * *p* < 0.05 d90 vs. Cont
**Ag**	**Median**	**25%**	**75%**	**H**
Control	0.03	0.02	0.06	H = 9.3, *p* = 0.01.
d0	0.1	0.03	0.155 #	MW (* *p* = 0.005 d0 vs. control). (# d90 vs. d0, *p* = 0.031)
d90	0.055	0.025	0.075	
**Sn**	**Median**	**25%**	**75%**	**H**
Control	0.045	0.02	0.095	H = 6.27, *p* = 0.43.
d0	0.11	0.04	0.20 *	MW (* d0 vs. Cont, *p* = 0.023).
d90	0.03	0.02	0.105 #	MW (# d90 vs. d0, *p* = 0.047).
**Zn^++^**	**Median**	**25%**	**75%**	**H**
Control	195	180	230	H = 5, *p* = 0.078
d0	245	208	275 *	MW (* *p* < 0.05 d0 vs. control).
d90	210	180	242	
**Cu^++^**	**Median**	**25%**	**75%**	**H**
Control	13.5	10.5	35.5	H = 1.01, *p* = 0.6, n.s
d0	15	11	31	
d90	13.5	10	19.5	
**Materials from Dental Titanium Alloys (Cr, Ni, Co)**				
**Al**	**Median**	**25%**	**75%**	**H**
Control	2.9	2.05	5.6	H = 4.6, *p* = 0.1, n.s.
d0	3	1.6	4.6	
d90	1.6	1.5	2.4 *	MW (* d90 vs. control, *p* = 0.029).
**Cr**	**Median**	**25%**	**75%**	**H**
Control	0.35	0.31	0.39	H=9.64, *p* = 0.008
d0	0.35	0.35	0.39	
d90	0.41	0.36	0.45 *,#	MW or Dunn’s.* *p* < 0.05 vs. cont, # *p* < 0.05 d90 vs. d0
**Co**	**Median**	**25%**	**75%**	**H**
Control	0.004	0.004	0.010	H = 4.97, *p* = 0.083, n.s.
d0	0.017	0.04	0.035 *	MW * *p* < 0.05 d0 vs. control.
d90	0.06	0.035	0.012	
**Ni**	**Median**	**25%**	**75%**	**H**
Control	0.055	0.04	0.10	H = 3.07, *p* = 0.21, n.s.
d0	0.09	0.08	0.16 *	MW (* *p* < 0.05, d0 vs. Cont).
d90	0.11	0.04	0.16	
**V**	**Media**	**S.E.M**		**F**
Control	0.04	0.003		F (2.50) = 2.73, *p* = 0.07, n.s.
d0	0.031	0.004 *		Bonferroni (*p* = 0.043, alpha (α) = 0.05, beta (β) = 0.42). * *p* < 0.05 vs. control
d90	0.041	0.0035 #		(# *p* < 0.05, d90 vs. d0)
	* *p* < 0.05 vs. Cont		# *p* < 0.05 d90 vs. d0	n.s: non significant effect (*p* > 0.05, n.s).

Percentiles analysis for heavy metals/oligoelement levels in Kruskal-Wallis (H) between patients with long-term titanium implant and dental fillings after 90 days of supplementation (d90, *n* = 16) as compared with their own basal levels (d0: before any supplementation, *n* = 16) and untreated (non-supplemented) controls without dental materials (control, *n* = 21). All heavy metals and oligoelements were expressed as μg/g of hair. H is the Krukal-Wallis analysis and F is ANOVA data. MW = Mann Whitney, S.E.M = standard error media; Control: controls without dental materials and non-supplemented (*n* = 21); d0: patients with long-term titanium implants and dental amalgam fillings restorations before any supplementation (d0, *n* = 16); d90: patients with long-term titanium implants and amalgams after 90 days of supplementation (*n* = 16); n.s: not significant effect, *p* > 0.05. * *p* < 0.05 vs. Control; # *p* < 0.05 d90 vs. d0.

**Table 4:** Percentiles for oligoelements involved in metabolic functions in patients with long-term titanium implant and dental fillings after 90 days of supplementation (d90, *n* = 16) and their basal levels (d0: before any supplementation, *n* = 16) and non-supplemented controls (control: without dental materials and non-supplemented, *n* = 21). All heavy oligoelements were expressed as μg g/g of hair.

**Se**	**Median**	**25%**	**75%**	**H**
Control	0.66	0.59	0.73	H = 10.91, *p* = 0.004.
d0	0.6	0.47	0.67 *	MW (* d0 vs. control, *p* = 0.05).
d90	0.55	0.48	0.62 * #	MW (# d90 vs. d0, *p* = 0.039; * *p* < 0.05 vs. cont).
**Mo**	**Median**	**25%**	**75%**	**H**
Control	0.034	0.0140	0.0032	H = 14.5, *p* < 0.001.
d0	0.022	0.0079	0.023 *	MW or Dunn’s (* *p* < 0.05 d0 vs. control).
d90	0.020	0.0084	0.150 *	MW or Dunn’s: * *p* < 0.05 d90 vs. control
**Mn^++^**	**Median**	**25%**	**75%**	**H**
Control	0.075	0.06	0.10	H = 5.42, *p* = 0.066, n.s.
d0	0.085	0.04	0.11	
d90	0.115	0.07	0.18 * #	MW (* d90 vs. control, *p* = 0.05); MW (d90 vs. d0, *p* < 0.05; * *p* < 0.05 vs. cont)
**Li**	**Median**	**25%**	**75%**	**H**
Control	0.008	0.006	0.012	H = 1.45, *p* < 0.001.
d0	0.005	0.0045	0.0075 *	MW (* d0 vs. control, *p* = 0.03).
d90	0.023	0.010	0.010 * #	MW (* d90 vs. control, *p* < 0.05); MW (# d90 vs. d0, *p* = 0.05).
**Ge**	**Median**	**25%**	**75%**	**H**
Control	0.031	0.024	0.033	H = 13.1, *p* = 0.01.
d0	0.024	0.021	0.032	MW (* d0 vs. control, *p* = 0.1, n.s).
d90	0.023	0.032	0.035 #	MW or Dunn’s (# *p* < 0.05 d90 vs. d0).
**S**	**Median**	**25%**	**75%**	**H**
Control	47700	47200	49250	H = 3.97, *p* = 0.13, n.s.
d0	46900	46350	47600 *	MW (* d0 vs. control, *p* < 0.05).
d90	46850	45600	49950	
**P**	**Median**	**25%**	**75%**	**H**
Control	185	155	197	H = 8.88, *p* = 0.012.
d0	153	134	160 *	MW (* d0 vs. control, *p* < 0.05).
d90	170	154	179 #	MW (# d90 vs. d0, *p* = 0.004).
**I**	**Median**	**25%**	**75%**	**H**
Control	0.58	0.37	2.05	H=3.67, *p* = 0.15, n.s
d0	0.39	0.29	0.5	
d90	0.47	0.31	0.77	
**Ca^++^**	**Median**	**25%**	**75%**	**H**
Control	488	285	705	H = 1.37, *p* = 0.5, n.s
d0	785	410	1262	
d90	676	380	1060	
**Sr**	**Median**	**25%**	**75%**	**H**
Control	2.7	0.93	6.55	H = 4.41, *p* = 0.11, n.s
d0	7	2.97	11.7	d0 vs. control, *p* = 0.064, n.s.
d90	11.81	1.9	17.75	d90 vs. control, *p* = 0.099, n.s
**B**	**Median**	**25%**	**75%**	**H**
Control	0.5	0.41	0.87	H = 1.5, *p* = 0.46, n.s.
d0	0.63	0.56	1.1	
d90	0.71	0.52	0.8	
**Na^+^**	**Median**	**25%**	**75%**	**H**
Control	35	14.5	73.25	H = 6, *p* =0.05.
d0	62.5	52	140 *	MW or Dunn’s (* d0 vs. control, *p* = 0.046).
d90	48	33	75	d90 vs. control, *p* = 0.1, n.s.
**K^+^**	**Median**	**25%**	**75%**	**H**
Control	13.5	4	31.5	H = 1.3, *p* = 0.52, n.s
d0	5.5	3.5	44	
d90	8.5	3	15	
**Mg^++^**	**Median**	**25%**	**75%**	**H**
Control	50	32.5	94	H = 3.63, *p* = 0.16, n.s.
d0	99	43.5	184.5	
d90	137	57	345	MW (d90 vs. control, *p* = 0.088, n.s).
**Rb**	**Median**	**25%**	**75%**	**H**
Control	0.015	0.0045	0.031	H = 2.72, *p* = 0.25, n.s.
d0	0.014	0.0040	0.019	
d90	0.011	0.0030	0.013	
**Fe^++^**	**Median**	**25%**	**75%**	**H**
Control	6.7	6.2	7.7	H = 3.7, *p* = 0.15, n.s.
d0	6.6	6.4	7.4	
d90	7.7	6.5	8.4 #	MW (d90 vs. d0, # *p* = 0.022).
	* *p* < 0.05 vs. control		# *p* < 0.05 d90 vs. d0	

* *p* < 0.05 vs. control; # *p* < 0.05 d90 vs. d0; controls (without dental materials and non-supplement; control, *n* = 21); d0: patients with long-term titanium implants and dental amalgam fillings restorations before any supplementation (d0, *n* = 16); d90: patients with long-term titanium implants and dental amalgams after 90 days of supplementation (d90, *n* = 16); (n.s: not significant effect, *p* > 0.05; * *p* < 0.05 vs. Control; # *p* < 0.05 d90 vs. d0).

**Table 5:** Decreased lead (Pb) levels after 90 days of supplementation as compared with baseline levels.

**Ba**	**Median**	**25%**	**75%**	**H**
Control	0.17	0.1	0.33	H = 7.73, *p* = 0.021.
d0	0.54	0.25	0.84 *	MW (* d0 vs. control, *p* = 0.05).
d90	0.28	0.2	0.36	MW (d90 vs. control, *p* = 0.11, n.s).
**Pb**	**Median**	**25%**	**75%**	**H**
Control	0.11	0.07	0.3	H = 3.41, *p* = 0.18, n.s.
d0	0.14	0.09	0.21	
d90	0.085	0.05	0.14 #	MW (# d90 vs. d0, *p* = 0.047).
**Cd**	**Median**	**25%**	**75%**	**H**
Control	0.009	0.009	0.010	H = 4.73, *p* = 0.094, n.s.
d0	0.009	0.009	0.009	
d90	0.009	0.009	0.009	MW (d90 vs. control, *p* = 0.08, n.s).
**Sb**	**Median**	**25%**	**75%**	**H**
Control	0.01	0.01	0.018	H = 3.5, *p* = 0.16, n.s.
d0	0.01	0.01	0.01	
d90	0.01	0.01	0.01	
**As**	**Median**	**25%**	**75%**	**H**
Control	0.038	0.01	0.018	H = 0.9, *p* = 0.62, n.s.
d0	0.028	0.022	0.042	
d90	0.028	0.023	0.052	
	* *p* < 0.05 vs. Cont		# *p* < 0.05 d90 vs. d0	

These tables show percentile values (median, 25%, and 75%) in Kruskal Wallis analysis for several heavy metals/oligoelements (Hg^++^, Ag, Sn, Zn^++^, Cu^++^, Al, Cr, V, Co, and Ni), metabolic oligoelements (Se, Mo, Mn^++^, Li, Ge, S, P, I, Ca^++^, Sr, B, Na^+^, K^+^, Mg^++^, Rb, B, and Fe^++^), and metals of environmental exposure in Table 6 (Ba, As, Pt, Sb, Tl, To, Cd, Be, Bi, Zr, Pb, Cd, As, and U). The ANOVA data for V are shown by mean values ± S.E.M [the root square divided by n; n was the size sample; *n* = 16 (d90), *n* = 16 (d0), *n* = 21 controls]. Post hoc differences were evaluated by the Mann Whitney or Dunn’s method; Control: controls without dental materials and non-supplemented (*n* = 21); d0: patients with long-term titanium implants and dental amalgam fillings restorations (d0, *n* = 16); d90: patients with long-term titanium implants and dental amalgams after 90 days of supplementation (*n* = 16); n.s: not significant effect, *p* > 0.05). * *p* < 0.05 vs. Control; # *p* < 0.05 d90 vs. d0).

**Table 6:** Effects on Se/Hg^++^, Se/Ag, Se/Al, Se/Pb, Mo/Hg^++^, Na+/K+ before/after nutritional supplementation and untreated controls.

**Se/Hg^++^ Ratio**	**Median**	**25%**	**75%**	**H**
Control	2.21	1.6	3.2	H = 31.42, *p* < 0.001.
d0	0.23	0.17	0.31 *	MW (* d0 vs. control, *p* < 0.001).
d90	0.28	0.24	0.56 * #	MW (# d90 vs. d0, *p* = 0.05. * *p* < 0.05 vs. cont).
**Se/Ag ratio**	**Median**	**25%**	**75%**	**H**
Control	22.3	12.26	37.7	H = 6.25, *p* = 0.044
d0	7.16	4.12	20.5	MW (* d0 vs. control, *p* = 0.04).
d90	11	6.8	18.2 *	MW (* d90 vs. control, *p* = 0.032).
**Se/Al ratio**	**Median**	**25%**	**75%**	**H**
cont	0.26	0.1	0.39	H = 3.76, *p* = 0.15, n.s.
d0	0.14	0.07	0.29	
d90	0.34	0.19	0.36 #	MW (# d90 vs. d0, *p* = 0.05).
**Se/Pb**	**Median**	**25%**	**75%**	**H**
Control	4.57	1,79	9.1	H = 1.12, *p* = 0.57, n.s
d0	3.81	2.2	6.9	
d90	5.72	3.76	8.24	
**Mo/Hg^++^**	**Median**	**25%**	**75%**	**H**
Control	0.018	0.011	0.03	H = 13.51, *p* = 0.001
d0	0.0089	0.0067	0.011 *	MW or Dunn’s, * d0 vs. Cont, *p* = 0.001
d90	0.026	0.011	0.112 * #	MW or Dunn’s, # d90 vs. d0, *p* < 0.001, * *p* < 0.05 vs. control
**Na^+^/K^+^**	**Median**	**25%**	**75%**	**H**
Control	3.57	4.46	0.94	H = 2.59, *p* = 0.2, n.s.
d0	7.74	16.3	0.82 *	MW (* *p* < 0.05, d0 vs. control).
d90	8	13.6	0.79	MW (d90 vs. control, *p* = 0.065, n.s).
	* *p* < 0.05 vs. control		# *p* < 0.05 d90 vs. d0	

Control: controls without dental materials and non-supplemented (*n* = 21); d0: patients with long-term titanium implants and dental amalgam fillings restorations (d0, *n* = 16); d90: patients with long-term titanium implants and dental amalgams after 90 days of supplementation (*n* = 16); n.s: not significant effect, *p* > 0.05; * *p* < 0.05 vs. Control; # *p* < 0.05 d90 vs. d0.

## Supplementary Materials

The following are available online at https://www.mdpi.com/2076-3921/8/4/101/s1, Table S1: correlations between Se, Mo and heavy metal ratios, Table S2: correlations between heavy metals and oligoelements (r Spearman).
