# Supplementary material for: The Long-Term Algae Extract (Chlorella and Fucus sp) and Aminosulphurate Supplementation Modulate SOD-1 Activity and Decrease Heavy Metals (Hg++, Sn) Levels in Patients with Long-Term Dental Titanium Implants and Amalgam Fillings Restorations
Source: Antioxidants (Basel). 2019 Apr 16;8(4):101. doi: 10.3390/antiox8040101 (PMC6523211; doi:10.3390/antiox8040101)
Supplement: Supplementary file 1 [file antioxidants-08-00101-s001.pdf]

APPENDIX (Supplementary files)

**-CONSORT 2017 guidelines**

*Title: The Long-Term Algae Extract (Chlorella and Fucus sp) and Aminosulphurate Supplementation Modulate SOD-1 Activity and Decrease Heavy Metals ($Hg^{++}$, Sn) Levels in Patients with Long-Term Dental Titanium Implants and Amalgam Fillings Restorations.*

*Background*: Humans are exposed to pollutants, xenobiotics, and heavy metals, which can be accumulated in the body when detox mechanisms are defective. Heavy metals can affect metallothionein and glutathione levels (GSH: reduced form) as well as SuperOxide Dismutase-1 (SOD-1) enzymatic activity. Selenium (Se) is a crucial element for heavy metal removal by conjugation with glutathione reduced form (GSH).
*Aim*: we evaluated whether 90 days of nutritional supplementation (d90, n=16) with *Chlorella vulgaris* and *Fucus* sp extracts in conjuntion with aminosulfurate nutraceuticals could detox heavy metal levels in patients with long-term titanium dental implants (average: 3, average: 12 years) and/or amalgam fillings (average: 4, average: 15 years) compared to baseline levels (d0: before any supplementation. n=16) and untreated controls (without dental biomaterials) of similar age (Control, n=21).

*Hypothesis*
The long-term algae exract (*Chrorella vulgaris c*and *Fucus* sp) and aminoazuphrates phytonaturals supplementation could detoxificate heavy metal levels in patients with long-term dental titanium implants and dental amalgam fillings restorations.
The long-term nutritional supplementation with these phytonaturals could prevent certain oligoelement deficits in these patients.

*Trial design*

***Material and methods:*** the following heavy metals were quantified in hair samples as an index of chronic heavy metal exposure before and after 90 days supplementation using ICP-MS and expressed as µg/g of hair (Al, Hg, Ba, Ag, Sb, As, Be, Bi, Cd, Pb, Pt, Tl, Th, U, Ni, Sn, Ti); we also measured several oligoelements ($Ca^{++}$, $Mg^{++}$, $Na^{++}$, $K^{+}$, $Cu^{++}$, $Zn^{++}$, $Mn^{++}$, Cr, V, Mo, B, I, P, Se, Sr, P, Co, $Fe^{++}$, Ge, Rb, Zr).

**Results**: The algae and nutraceutical supplementation during 90 consecutive days decreased $Hg^{++}$, Ag, Sn, Pb, at 90 days as compared to baseline levels; these mercury levels at 90 days were lower than untreated controls.
The suplementation contribute to reduce heavy metal levels. We found increased Li and Ge levels after 90 days of supplementation in patients with long-term dental titanium implants and amalgams. They (d90) increased $Mn^{++}$, P, and $Fe^{++}$ levels as compared with their basal levels (d0: before any supplementation) and untreated controls (control). Finally, decreased SOD-1 activity (saliva) was observed after 90 days of supplementation as compared with basal levels (before any supplementation, d0), suggesting antioxidant effects. Conversely, increased SOD-1 activity were observed after 90 days as compared with untreated controls (cont).

*Participants: elegible criteria for participants*
d0 (n=16), d90 (n=16), controls (n=21).

*Interventions*
**Controls**: untreated patients were selected after clinical examination together phone call (n=21).
and interview by a dentist from CIROM (Murcia).
They don't have dental materials in mouth and absence of periodontal diseases.
They did not receive nutritional supplementation.
Hair samples were collected for ICP-MS analysis of heavy metals and oligoelements (control, n=21).
The fish consumption was 1-2 times by week in all recruited patients, including controls.

**d0**: the average number of dental amalgam fillings were four and 3 dental titanium implant alloys.
They have long-term dental amalgam filling at least 10 years (average: 15 years in mouth) and long-term dental titanium alloys at least for 10 years (average: 12 years).
All dental amalgam were progressively restored by composites (Bisphenol A free). They don`t have metabolic alterations or periodontal disease.
Their fish consumption were 1-2 times by week.
Hair samples were collected before nutritional supplementation (d0, n=16) for heavy metals /oligoelements determination by ICP-MS (Dr. DATA, USA).

**d90 patients:** The average number of dental amalgam fillings were four dental fillings and three dental titanium implant alloys.
All selected patients have long-term dental amalgam fillings at least 10 years (average: 15 years in mouth) and long-term titanium dental alloys (average: 12 years).
They received nutritional supplementation during 90 days with these formulations:
GREEN-FLOR (2-0-2; 4 capsules/day: *Chlorella and Fucus* algae extract), ERGYTAURINE (1-0-1; 2 capsule/day) and ERGYLIXIR (d90, n=16).
Their hair samples analysis were compared after 90 days (d90) with their baseline levels (d0: before any supplementation).
Hair samples were collected after nutritional supplementation (d90, n=16) for heayy metals /oligoelements quantification by ICP-MS (Dr. DATA, USA).
Their fish compsumption were 1 or 2 times/week.
They don`t have metabolic alterations or periodontal disease.

*Exclusion criteria* (for all study groups)
-Periodontal disease or gingivitis,
-presence of bacterial (plaque).
- orthodontic devices.
-the use of removable partial denture.
-fish consumption higher than 2 times by week.

*Methods for biochemical evaluation*
Heavy metals/oligoelements were measured by hair ICP-MS at baseline levels (d0: before any supplementation), as well as at 90 days of supplementation (d90); we also included =21 controls without dental materials and non supplemented for ICP-MS analysis here.
SOD-1 actitivy was measured following our previous protocols.

Patients are from Murcia (Spain) and they visit CIROM Clinic for rutinatry evaluation.
ICP-MS analysis in hair samples were done by Dr. DATA laboratory (USA).
Elegibility for patients are described in the mean text.
There were not care providers involved in the present study.

*Outcomes*

Results were evaluated by ICP-MS analysis in hair samples before any nutritional supplementation (d0), as well as after 90 days of supplementation (d90); untreated controls (without dental materials and non supplemented, n=21) were also included in this study.
We compared a plethora of heavy metals/oligoelements between all study groups (n=37 patients and 52 hair samples).
SOD-1 activity (salive) were quantified in these 43 samples.
Correlations between variables were stablished by r Spearman test.

*Sample size*

The size sample was stimated by G-Power (**http://www.gpower.hhu.de/en.html).** n=37 patients and 52 hair samples).

*Allocation concealment mechanism and sequence generation: implementation and blinding.*

Mechanism used to implement the random allocation sequence have been done by computer; Interventions were assesed in patients for ICP-MS (hair) and SOD-1 determination (saliva). Dentist from CIROM (Murcia) generated the random allocation sequence, and we enrolled 37 participants after calling 152 patients (52 analyzed hair samples). These untreated controls (n=21), don`t have long-term dental titanium and dental amalgam filling/s restorations; they don`t receive nutritional supplementation. Dentist cooded all hair samples until ICP-MS quantification by researcher. Saliva samples were also blinded and cooded for SOD-1 activity determination

*Statistical methods.*

Levene test: identify homogeneity of variance
Kruskall Walis (KW) and pos Hoc Mann-Whitney test (MW) for non parametric data
ANOVA and post Hoc Bonferroni for homogeneity of variance (vanadium only).
r Spearman correlation task were used for multiple correlation between heavy metals and oligoelements.
Statistical analysis for all heavy metals/oligoelements were quantified in n = 21 controls, n = 16 d0 patients and n = 16 d90 patients.

*Results.*

Cont (n = 21), d0 (n = 16), d90 (n = 21).
Kruskal Walis values for each results are described in the text (see material and method section).
ANOVA for Vanadium are showed here.
Briefly, increased Li and Ge, $Mn^{++}$, P and $Fe^{++}$ levels were observed here (d90 vs cont).
Increased SOD-1 activity (salive, d0 vs cont)

Decreased SOD-1 activity (d90 vs d0)

*Recruitment*

Patients were recruited from January 2015 to January 2016 (2 year).

During the follow-up period ( june to december) of last year all samples were collected for biochemical evaluation. Dentist collected all hair samples for ICP-MS analysis and saliva samples for SOD-1 activity.

*Baseline data*

*Numbers analyzed*

d0 (n=16)

d90 (n=16)

Cont (n=21).

*Clinical characteristic of patients*

| | Cont | d0 and d90 |
|---|---|---|
| Age (years) | 42-70 | 49-68 |
| Sex (female) | 40% | 60% |
| Smoking status: | 14% | 7 % |

*Harms*

There were no adverse effects; the long-term supplementation with algae and aminoazuphrates is safe and not toxic for humans.

*Discussion*

*Limitations*

The absence of placebo group is a limitation. However, we added untreated controls (without dental materials and non-supplemented) for statistical comparisons. This pilot study has been done with Caucasian population (Spaniards) and must be validated in other ethnic group.

*Generability.*

The external validity of these findings should be confirmed with other ethnias; the present pilot study was performed in spaniers (Caucasian population). There is no care providers.

*Interpretation*

The long-term algae extract and aminosulphurate supplementation promote detoxification of heavy metals in patients with long-term titanium alloys and dental amalgams restorations.

The long-term with algae extract and aminosulfurates for 90 consecutive days decreased certain heavy metal levels ($Hg^{++}$, Ag, Sn, Pb) as compared with their baseline levels (d0: before any supplementation). However $Hg^{++}$ and Sn reductions were observed after 90 days of supplementation as compared with non supplemented controls (without dental materials). The dental restoration

using carbon active (nasal filters) in conjunction with long-term nutritional supplementation contribute to detoxificate heavy metals in these patients.

*Fundings.*

Funds # 20151602 from CIROM (Murcia, Spain).
Funds supported by Nutergia Laboratory (Basque Country, Spain). The funder Nutergia had not role in the design of the study; in the collection, analysis or interpretation of data; in the writing of the manuscript or in the decision to publish the results. APC supported by Nutergia Laboratories (Basque Country).

**Appendix: correlations between heavy metals and oligoelements**

**Table S1: correlations between Se, Mo and heavy metal ratios.**

| | | | | |
|---|---|---|---|---|
| | **Se (d90)** | **Hg++ (d90)** | **Ni (d90)** | **P (d90)** |
| **Se/Hg++ (d90)** | r=-0.76,p=0.004 | r=-0.93, p=0.0000002 | r=-0.59, p=0.005 | r=-0.76, p=0.005 |
| | **Na+ (d90)** | **Ba (d90)** | **Rb (d90)** | |
| **Se/Hg++ (d90)** | r=-0.56, p=0.004 | r=0.58, p=0.016 | r=-0.5, p=0.045 | |
| | **Hg++ (d0)** | **Ge (d0)** | | |
| **Se/Hg (d0)** | r=-0.87, p=0.0005 | r=-0.74, p=0.0005 | | |
| | **Hg++ (d90)** | **Mo (d90)** | | |
| **Se/Ag (d90)** | r=-0.54, p=0.028 | r=-0.93, p=0.0000002 | | |
| | **Hg++ (d90)** | **Na+/K+ (d0)** | | |
| **Se/Ag (d0)** | r=-0.9, p=0.0000002 | r=-0.54, p=0.028 | | |
| | **Se (d90)** | **Se/Hg++ (d90)** | **Se/Al (d0)** | **V (d90)** |
| **Se/Al (d90)** | r=0.73, p=0.0007 | r=0.62, p=0.009 | r=0.48, p==.05 | r=0.53, p=0.03 |
| | **Ba (d90)** | **Na+ (d90)** | **B (d90)** | **Ag (d90)** |
| **Se/Al (d90)** | r=-0.6, p=0.01 | r=-0.56, p=0.004 | r=0.58, p=0.016 | r=-0.45, p=0.011 |
| | **Ag (d0)** | **Ge (d0)** | **Sn (d0)** | **Fe++ (d0)** |
| **Se/Al (d0)** | r=-0.6, p=0.01 | r=0.5, p=0.05 | r=-0.56, p=0.009 | r=-0.6, p=0.01 |
| | **Pb (d90)** | **Se/Pb (d90)** | **Pb (d0)** | **Zn++ (d90)** |
| **Se/Pb (d90)** | r=-0.84, p=0000002 | r=0.69, p=0.03 | r=-0.75, p=0.0005 | r=0.53, p=0.036 |
| | **Pb (d0)** | **Se/Pb (d0)** | **Ba (d0)** | |
| **Se/Pb (d0)** | r=-0.95, p=0.0000002 | r=0.69, p=0.03 | r=0.59, p=0.01 | |

| | Mo/Hg++ (d0) | Hg++ (d90) | Se/Hg++ (d90) | Mo (d90) |
|---|---|---|---|---|
| **Mo/Hg++ (d90)** | r=0.57, p=0.039 | r=-0.88 p=0.0000002 | r=0.6, p=0.021 | r=-0.58, p=0.041 |

| | Mo (d0) | Fe++ (d0) | Mo/Hg++ (d90) | Se/Hg++ (d0) | Ge (d0) |
|---|---|---|---|---|---|
| **Mo/Hg++ (d0)** | r=0.54, p=0.05 | r=0.6, p=0.02 | r=0.88, p=0.0000002 | r=0.56, p=0.003 | r=-0.64, p=0.02 |

| | Fe++ (d0) | Mo (d0) |
|---|---|---|
| **Mo/Fe++ (d0)** | r=0.6, p=0.025 | r=-0.53, p=0.05 |

| | Li (d90) | Cr (d90) |
|---|---|---|
| **Na+/K+ (d90)** | r=0.53, p=0.05 | r=-0.55, p=0.043 |

**Table S2: correlations between heavy metals and oligoelements (r Spearman).**

| *Other correlations (r Spearman) between heavy metals and oligoelements* | *r (Regression)* | *p (*p<0.05)* |
|---|---|---|
| *Hg++ x B* | 0.67 | 0.028 |
| *Hg++ (90d) x B (90d)* | -0.65 | 0.042 |
| *Hg++ (90d) x Ca++ (90d)* | 0.68 | 0.032 |
| *Se/Hg++ (90d) x B (90d)* | 0.75 | 0.01 |
| *Se/Ag (90d) x Pb (90d)* | -0.8 | 0.003 |
| *Se (90d) x Sn (90d)* | -0.81 | 0.024 |
| *Se x Cr* | 0.75 | 0.005 |
| *Se x P* | 0.63 | 0.049 |
| *Li x Se/Hg++* | 0.69 | 0.021 |
| *Li x Se/Li* | -0.98 | <0.001 |
| *Li (90d) x Se/Li (90d)* | -0.91 | <0.001 |
| *Se/Al x P* | -0.62 | 0.0021 |
| *Cr x B* | 0.75 | <0.01 |
| *Cr x Se* | 0.76 | <0.05 |
| *Cr x P* | 0.76 | 0.025 |
| *Cr (90d) x P (90d)* | 0.59 | 0.009 |
| *Fe++ (90d) x P (90d)* | 0.68 | 0.034 |
| *Fe++ (90d) x B (90d)* | 0.62 | 0.05 |
| *P (90d) x B (90d)* | 0.6 | 0.05 |

| | | |
|---|---|---|
| ***S x Sn*** | -0.66 | 0.04 |
| ***B x Sn*** | 0.68 | 0.026 |
| ***P x B*** | 0.67 | 0.05 |
| ***Sn x Pb*** | 0.67 | 0.05 |
| ***P x Fe++*** | 0.6 | 0.03 |
